# Supplementary material for: Improved methods for marking active neuron populations
Source: Nat Commun. 2018 Oct 25;9:4440. doi: 10.1038/s41467-018-06935-2 (PMC6202339; doi:10.1038/s41467-018-06935-2)
Supplement: Supplementary file 1 — Reporting Summary [file 41467_2018_6935_MOESM1_ESM.pdf]

nature research

Corresponding author(s): *Double-blind peer review submissions: write DBPR and your manuscript number here instead of author names.*

Reporting Summary

Nature Research wishes to improve the reproducibility of the work that we publish. This form provides structure for consistency and transparency in reporting. For further information on Nature Research policies, see Authors & Referees and the Editorial Policy Checklist.

Statistical parameters

When statistical analyses are reported, confirm that the following items are present in the relevant location (e.g. figure legend, table legend, main text, or Methods section).

n/a	Confirmed	
☐	☒	The exact sample size (*n*) for each experimental group/condition, given as a discrete number and unit of measurement
☐	☒	An indication of whether measurements were taken from distinct samples or whether the same sample was measured repeatedly
☐	☒	The statistical test(s) used AND whether they are one- or two-sided<br>*Only common tests should be described solely by name; describe more complex techniques in the Methods section.*
☒	☐	A description of all covariates tested
☒	☐	A description of any assumptions or corrections, such as tests of normality and adjustment for multiple comparisons
☐	☒	A full description of the statistics including central tendency (e.g. means) or other basic estimates (e.g. regression coefficient) AND variation (e.g. standard deviation) or associated estimates of uncertainty (e.g. confidence intervals)
☒	☐	For null hypothesis testing, the test statistic (e.g. *F*, *t*, *r*) with confidence intervals, effect sizes, degrees of freedom and *P* value noted<br>*Give P values as exact values whenever suitable.*
☒	☐	For Bayesian analysis, information on the choice of priors and Markov chain Monte Carlo settings
☒	☐	For hierarchical and complex designs, identification of the appropriate level for tests and full reporting of outcomes
☒	☐	Estimates of effect sizes (e.g. Cohen's *d*, Pearson's *r*), indicating how they were calculated
☐	☒	Clearly defined error bars<br>*State explicitly what error bars represent (e.g. SD, SE, CI)*

Our web collection on statistics for biologists may be useful.

Software and code

Policy information about availability of computer code

Data collection	ScanImage (MATLAB), Nikon Elements, Zeiss Zen
Data analysis	IGOR Pro, MATLAB, Prism, ImageJ

For manuscripts utilizing custom algorithms or software that are central to the research but not yet described in published literature, software must be made available to editors/reviewers upon request. We strongly encourage code deposition in a community repository (e.g. GitHub). See the Nature Research guidelines for submitting code & software for further information.

Data

Policy information about availability of data

All manuscripts must include a data availability statement. This statement should provide the following information, where applicable:

- Accession codes, unique identifiers, or web links for publicly available datasets
- A list of figures that have associated raw data
- A description of any restrictions on data availability

Source data from experiments in this study are available from the authors upon reasonable request.

Field-specific reporting

Please select the best fit for your research. If you are not sure, read the appropriate sections before making your selection.

☒ Life sciences ☐ Behavioural & social sciences ☐ Ecological, evolutionary & environmental sciences

For a reference copy of the document with all sections, see nature.com/authors/policies/ReportingSummary-flat.pdf

Life sciences study design

All studies must disclose on these points even when the disclosure is negative.

Sample size	No specific sample size calculations were performed. Generally, sample size was the maximum possible within reasonable time and resource allocation limits.
Data exclusions	No data excluded.
Replication	Multiple experimental replicates were performed for each type of measurement described, and were found to be generally consistent.
Randomization	No randomization was performed.
Blinding	No blinding was performed.

Reporting for specific materials, systems and methods

Materials & experimental systems

n/a	Involved in the study	
☐	☒	Unique biological materials
☐	☒	Antibodies
☐	☒	Eukaryotic cell lines
☒	☐	Palaeontology
☐	☒	Animals and other organisms
☒	☐	Human research participants

Methods

n/a	Involved in the study	
☒	☐	ChIP-seq
☒	☐	Flow cytometry
☒	☐	MRI-based neuroimaging

Unique biological materials

Policy information about availability of materials

Obtaining unique materials	Anti-CaMPARI-red monoclonal antibodies and the corresponding hybridomas generated during this study are available from the authors upon request. Moreover, the amino acid sequence of antibody variable regions is described in the manuscript.

Antibodies

Antibodies used	mouse anti-CaMPARI-red, generated by Genscript as part of this work<br>rabbit anti-FLAG, Sigma-Aldrich, F7425<br>goat-anti-rabbit Alexa Fluor 405, Invitrogen, A31556<br>goat-anti-mouse Alexa Fluor 647, Invitrogen, A21236<br>donkey-anti-mouse Alexa Fluor 405, Jackson Immunoresearch, 715-475-151<br>donkey-anti-rabbit Alexa Fluor 647, 711-605-152<br>HRP-conjugated horse-anti-mouse, Cell Signaling, 7076
Validation	Mouse anti-CaMPARI-red antibodies were validated using Western Blot with purified proteins and immunohistochemistry wiht cultured cells using positive and negative controls. Commercial antibodies were validated by the manufacturers for the applications used.

Eukaryotic cell lines

Policy information about cell lines

Cell line source(s)	HeLa cells from American Type Culture Collection (ATCC)
Authentication	No authentication performed.
Mycoplasma contamination	Cell lines were tested and found negative for mycoplasma.
Commonly misidentified lines (See ICLAC register)	None

Animals and other organisms

Policy information about studies involving animals; ARRIVE guidelines recommended for reporting animal research

Laboratory animals	Rattus norvegicus (rat), Sprague-Dawley, male, 9-12 weeks<br>Mus musculus (mouse), B6.129-Camk4tm1Gsc/Ieg, male, p15<br>Mus musculus (mouse), C57bl/6J, female, 7-12 weeks<br>Mus musculus (mouse), C57bl/6, male and female, 40-100 days<br>Rattus norvegicus (rat), Wistar, male and female, 4-7 days
Wild animals	None
Field-collected samples	None
