# Supplementary material for: Improved methods for marking active neuron populations
Source: Nat Commun. 2018 Oct 25;9:4440. doi: 10.1038/s41467-018-06935-2 (PMC6202339; doi:10.1038/s41467-018-06935-2)
Supplement: Supplementary file 3 — Supplementary Information [file 41467_2018_6935_MOESM3_ESM.pdf]

Supplementary Figures

CaMPARI1	1	DQLTEEQIAEFKEAFSLFDKDGDGTITTKELGTVMRSLGQNPTEAELQDMINEVDADGDG	60
CaMPARI2	1	DQLTEEQIAEFKEAFSLFDKDGDGTITTKELGTVMRSLGQNPTEAELQDMINEVDADGDG	60
CaMPARI1	61	TIDFPEFLTMMARKMKDTDSEEEIREAFRVFDKDGNGYISAAELRHVMTNLGEKLTDEEV	120
CaMPARI2	61	TIDFPEFLTMMARKMKDTDSEEEIREAFRVFDKDGNGYISAAELRHVMTNLGEKLTDEEV	120
CaMPARI1	121	DEMIREADIDGDGQVNYEEFVQMMTAKLECEKIYVRDGVLTGDIHMILLLEGNAHYRCDF	180
CaMPARI2	121	DEMIREADIDGDGQVNYEEFVVMMTAKLECEKIYVRDGVLTGDIHMILLLEGNAHYRCDF	180
CaMPARI1	181	RTTYKAKEKGVKLPGVHFVDHCIEILSHDKDYNKVKLYEYAVAHSGLPDNARRGGTGGSM	240
CaMPARI2	181	RTTYKAKEKGVKLPGVHYVDHTIEILSHDKDYNKVKIYEYAVAHSGLPDNARRGGTGGSM	240
CaMPARI1	241	VSAIKPDMKIKLRMEGNVNGHHFVIDGDGTGKPYEGKQTMDLEVKEGGPLPFAFDILTTA	300
CaMPARI2	241	VSAIKPDMKIKLRMEGNVNGHHFVIDGDGTGKPYEGKQTMDLEVKEGGPLPFAFDILTTA	300
CaMPARI1	301	FHYGNRVFVKYPDNIQDYFKQSFPKGYSWERSMTFEDGGICYARNDITMEGDTFYNKVRF	360
CaMPARI2	301	FHYGNRVFVKYPDNIQDYFKQSFPKGYSWERSMTFEDGGICYARSDITMEGDTFYNKVRF	360
CaMPARI1	361	YGTNFPANGPVMQKKTLKWMPSWTRSSRRKWNKTGHAVRAIGRLSS*	406
CaMPARI2	361	YGTNFPANGPVMQKKTLKWMPSWTRSSRRKFNKTGHALRAIGRLSSGGSGGS**DYKDDDDK**	420
CaMPARI2		(F391W) ...SSRRKWNKTGHALRAIGRLSS...	
CaMPARI2		(H396K) ...SSRRKWNKTGKALRAIGRLSS...	
CaMPARI2		(F391W-G395D) ...SSRRKWNKTDHALRAIGRLSS...	
CaMPARI2		(L398T) ...SSRRKWNKTGHATRAIGRLSS...	
CaMPARI2	421	GGSGGS**YPYDVPDYA**GGSGGGS**EQKLISEEDL**RT*	453

Supplementary Figure 1: Sequence alignment of CaMPARI1, CaMPARI2 and the 4 different affinity variants of CaMPARI2. The calmodulin domain is shaded grey, while the calmodulin-interacting peptide is underlined; the chromophore is shaded black. The yellow residues are the mutations between CaMPARI1 and CaMPARI2 outside of the calmodulin-interacting peptide. The cyan residues are the affinity mutations described in Table 1 and Table S3. Epitope tags (FLAG – HA – myc) are in bold.

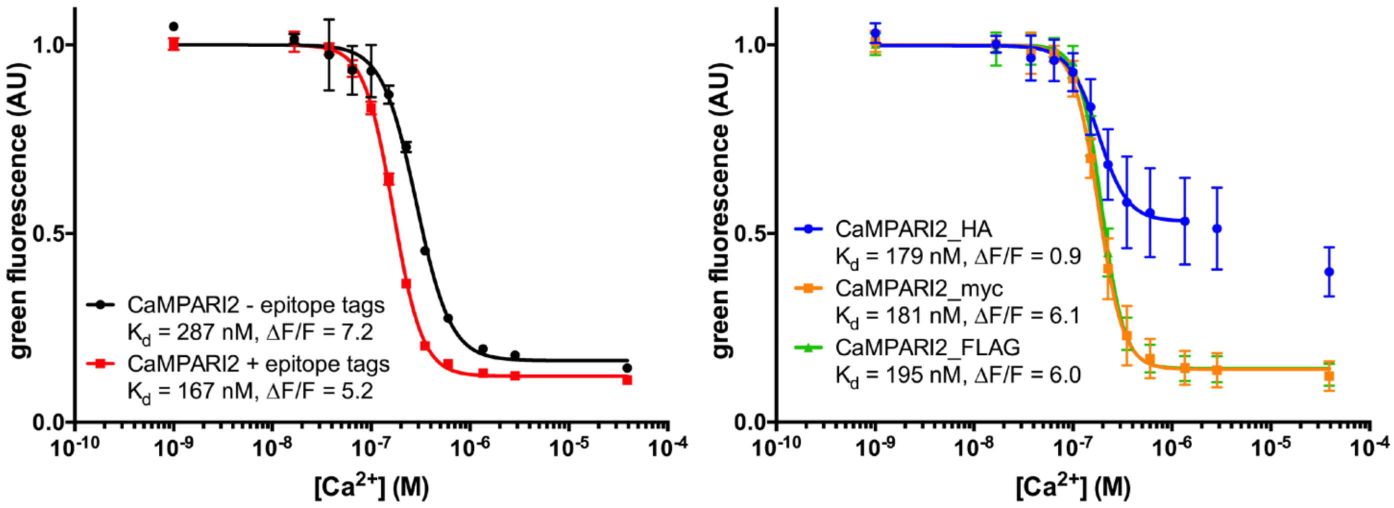

Supplementary Figure 2: Effect of adding epitope tags on the calcium affinity of CaMPARI2. Titration curves for calcium of CaMPARI2 with (*n* = 2) or without (*n* = 2) epitope tags attached (left) or CaMPARI2 with a single epitope attached (HA: *n* = 6, myc: *n* = 8, FLAG: *n* = 6) (right). The ΔF/F is the difference in green fluorescence intensity between high and low calcium conditions, divided by the green fluorescence intensity in high calcium conditions. Error bars are standard deviation.

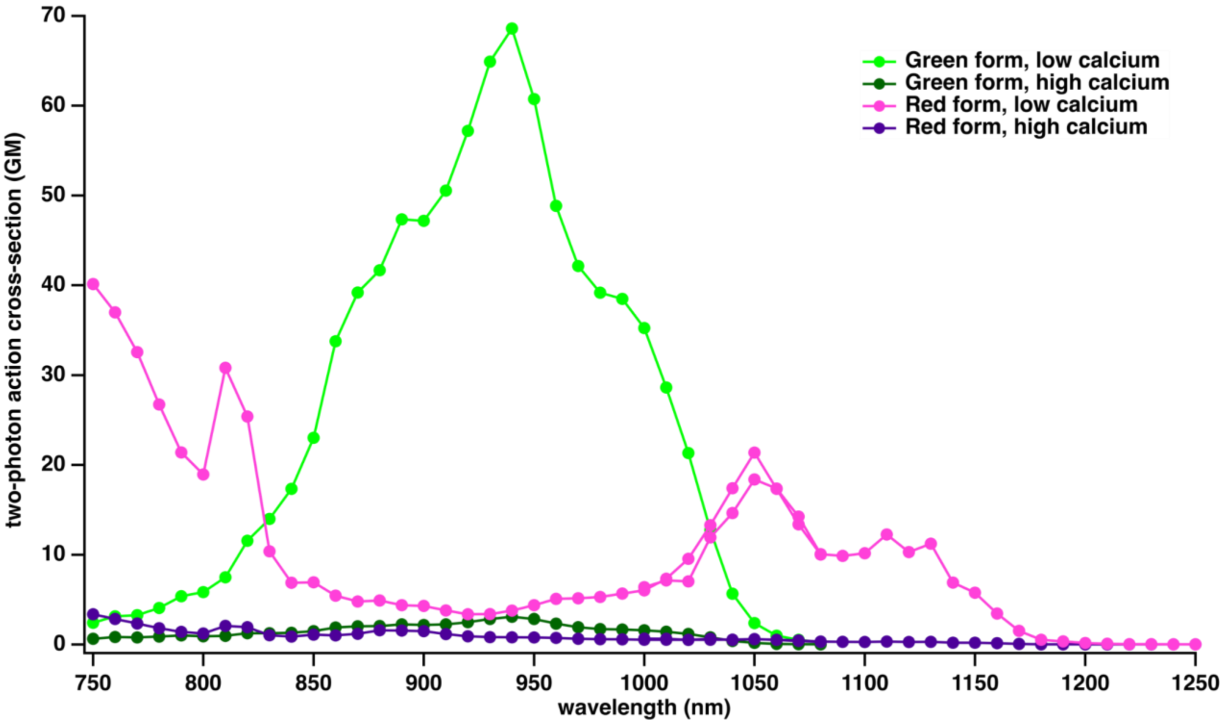

Supplementary Figure 3: Two-photon action cross-section of CaMPARI2. Spectra of the green and red state of CaMPARI2 in high and low calcium conditions are plotted. The peak at 810 nm that appears in the red species was not seen in the raw data and is probably an artifact of the reference spectrum[1] used to normalized for instrumental variation in the data acquisition.

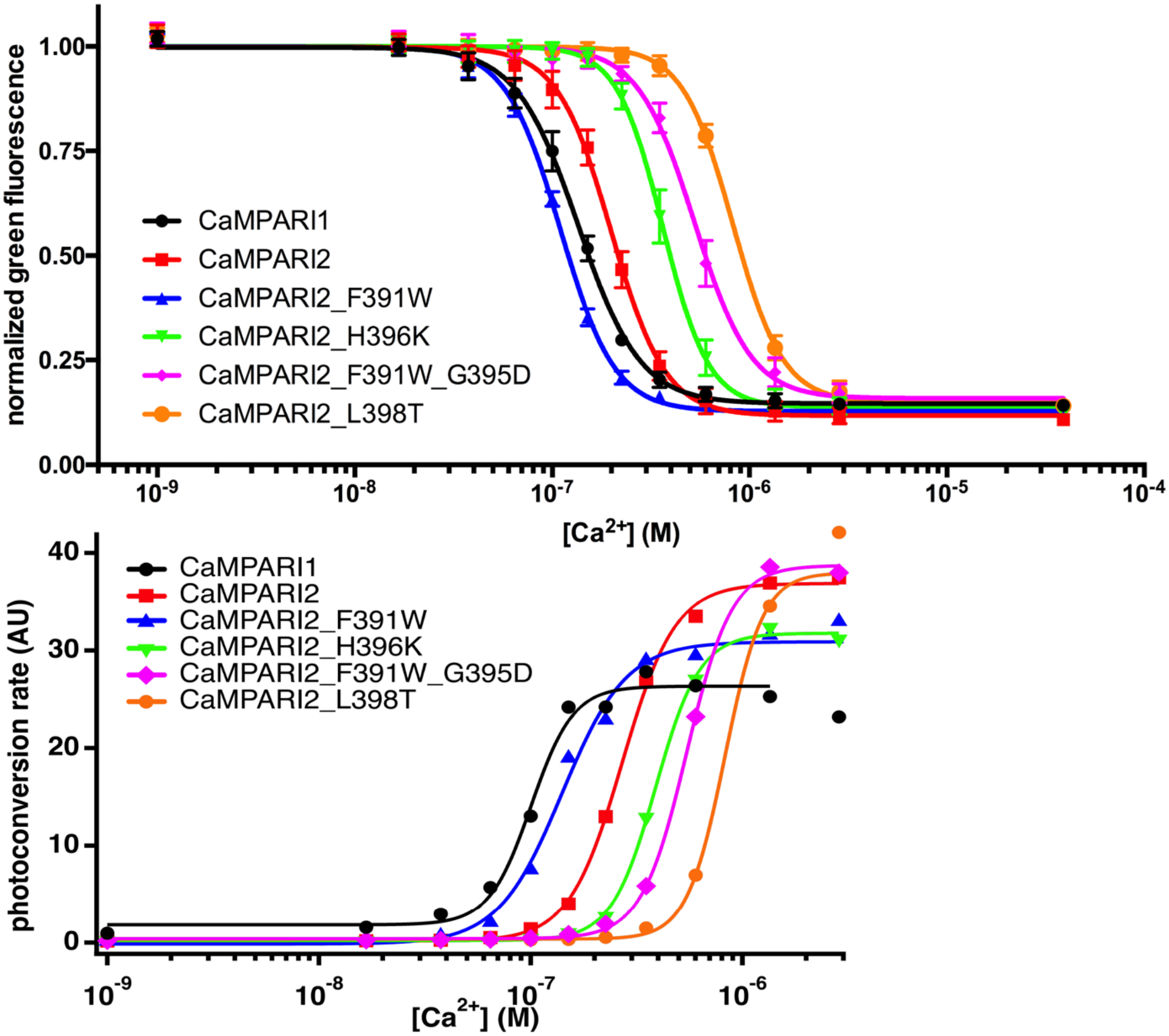

Supplementary Figure 4: Calcium titration of green fluorescence and photoconversion rate. The top graph represents the titration curves for Ca^{2+} of several CaMPARI variants, measuring the green fluorescence and normalized to 1 for low Ca^{2+} concentrations (n = 8, error bars are standard error). The bottom graph represents the rate of photoconversion for CaMPARI variants in different concentrations of Ca^{2+}.

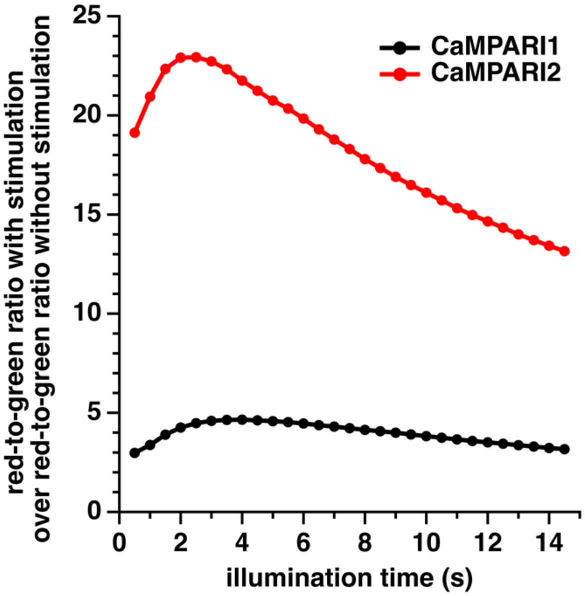

Supplementary Figure 5: Red-to-green contrast between high and low calcium photoconversion in neurons. The amount of photoconversion (as measured by the ratio between red and green CaMPARI) in high Ca^{2+} conditions (during field stimulation) versus low Ca^{2+} conditions (no field stimulation) is plotted against the cumulative time of 405 nm light delivered. Data derived from the data presented in Figure 1C, right panel.

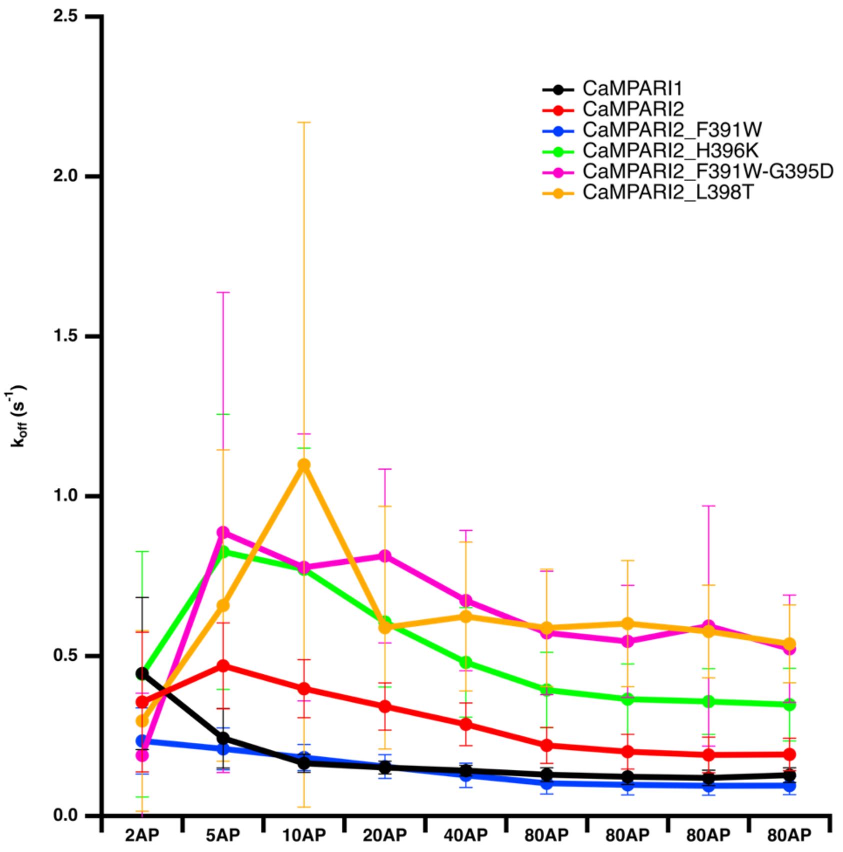

Supplementary Figure 6: Rate constant of calcium unbinding of CaMPARI2 in primary rat hippocampal neurons. The fitted rate constant of fluorescence change following cessation of stimulation (k_{off}, average of 6 trials) is plotted for each number of field stimulations. Error bars are standard deviation.

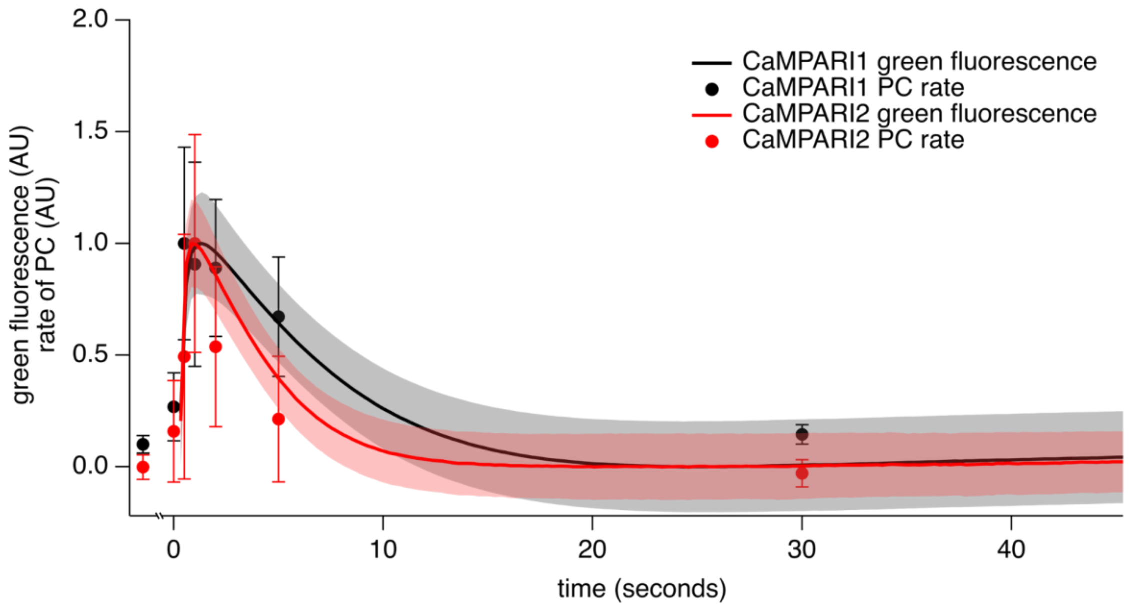

Supplementary Figure 7: Delay between stimulation and photoconversion light. The extent of photoconversion of CaMPARI1 and CaMPARI2 in cultured neurons are plotted as a function of the delay between field stimulation (20 AP at 80 Hz) and photoconversion light (395 nm, 250 ms at ~3 W/cm^2). Control measurements without stimulation are shown left of the t = 0 timepoint. Solid lines represent the normalized inverse of the green fluorescence during the imaging session, shaded areas represent standard deviation from between 220 and 408 cells, depending on the timepoint.

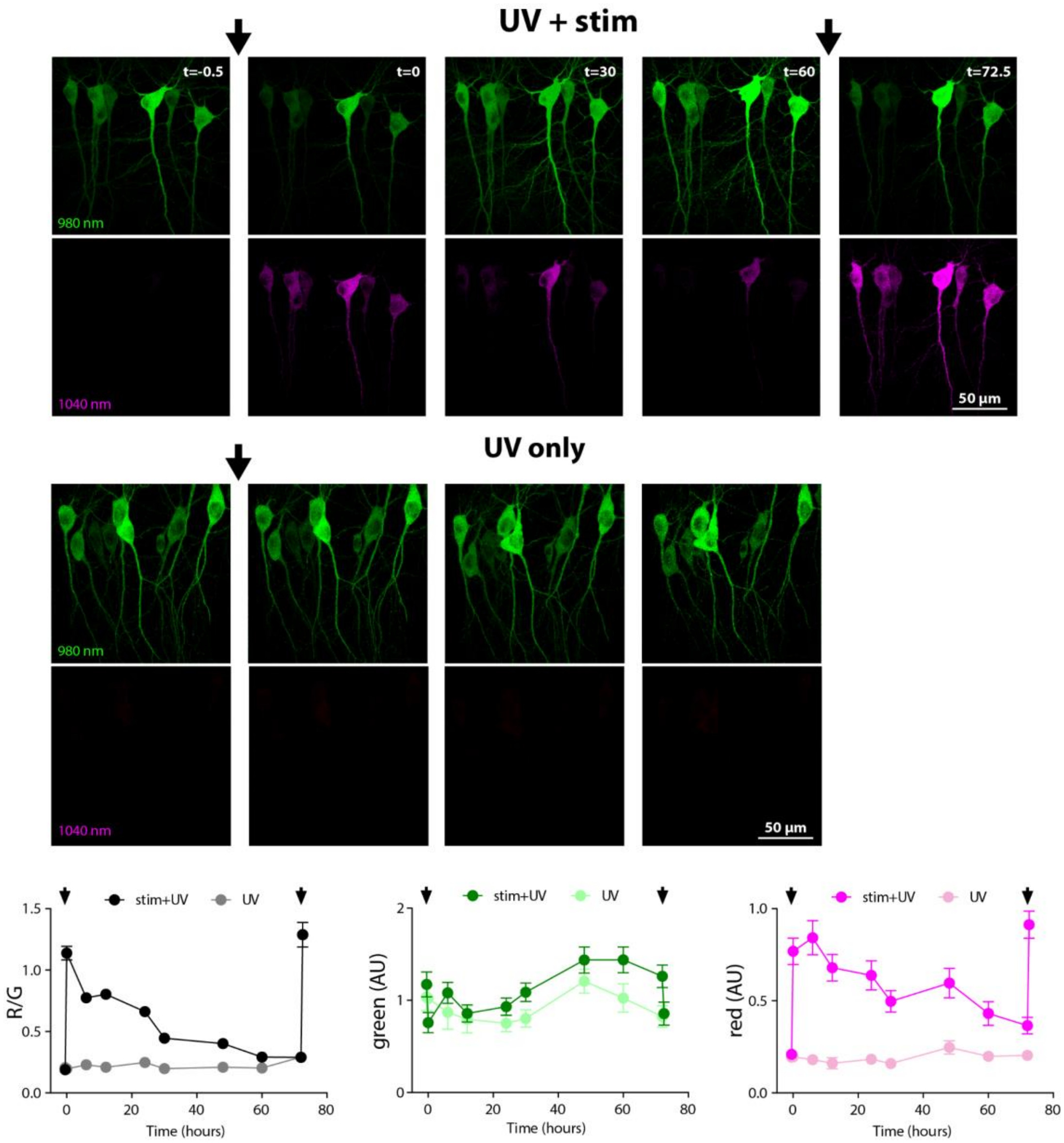

Supplementary Figure 8: Three day turnover of CaMPARI2 and re-conversion. Two-photon image stacks of CA1 neurons from rat hippocampal slice cultures starting 4 days after electroporation at DIV15 with DNA encoding CaMPARI2_F391W-L398V (no epitope tags) at the time points indicated (hours, t = 0 immediately after photoconversion). Arrows indicate when CaMPARI2 photoconversion was induced by combining strong electrical stimulation of synaptic inputs in the stratum radiatum (100 times at 100 Hz) with UV light (2 seconds, 395 nm, 16 mW mm^{-2}) applied with a 1 s delay from the start of stimulation (UV + stim; 24 neurons, 4 slices). Controls received the same UV without electrical stimulation (UV only; 21 neurons, 3 slices). Values are mean ± standard error of the mean.

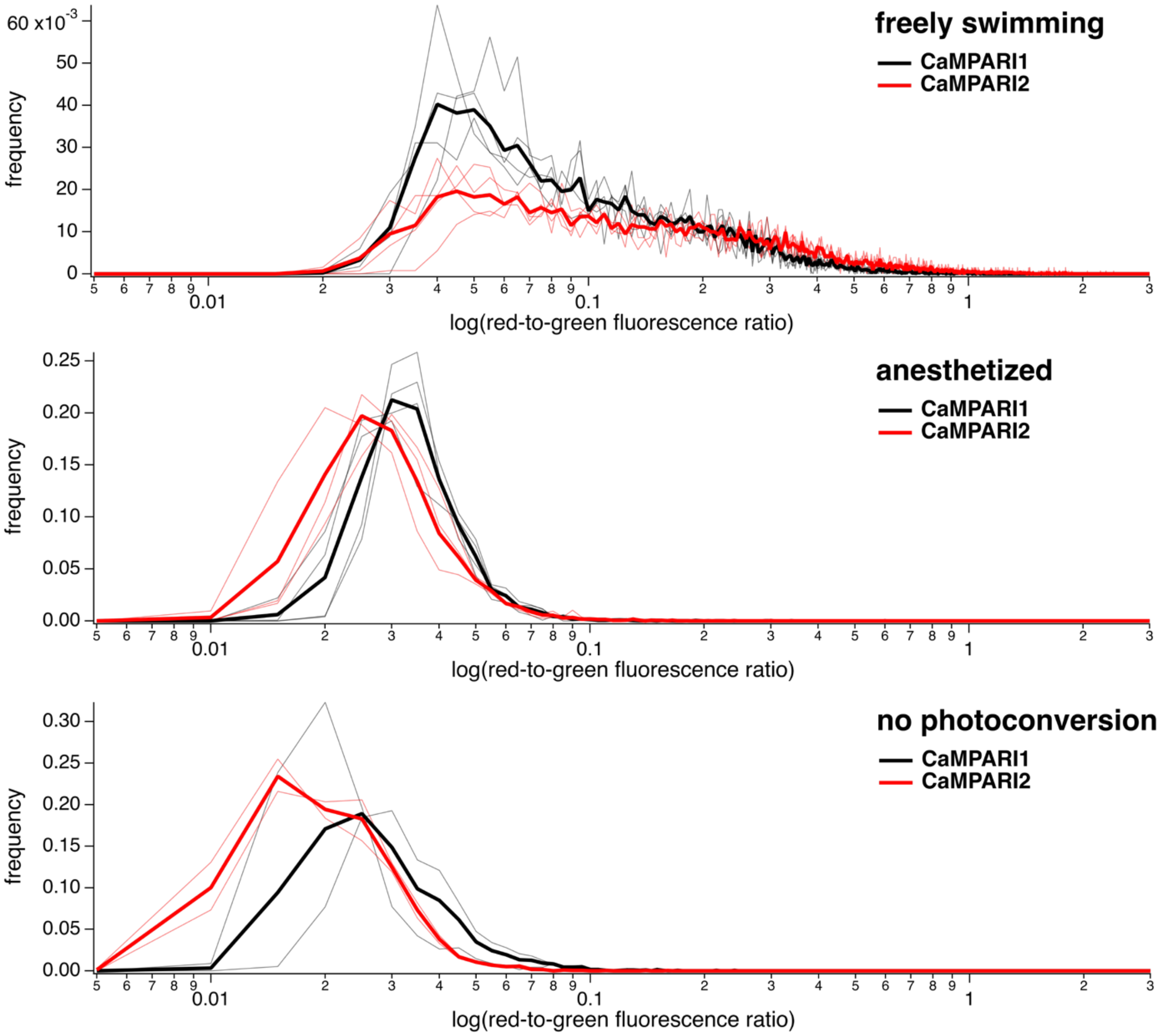

Supplementary Figure 9: Histograms of the red-to-green fluorescence ratio in zebrafish. Fish were photoconverted in the absence (top graph) or presence (middle graph) of the anesthetic tricaine or not photoconverted (bottom graph). Thin lines represent histograms of the red-to-green ratio of all cells from individual fish, thick lines are the histogram of all measured cells of all fish for the given condition. Note the log scale on the x-axis, intended to draw the focus on the low red-to-green ratios, but thereby obscuring the difference between CaMPARI1 and CaMPARI2 at higher red-to-green fluorescence ratios.

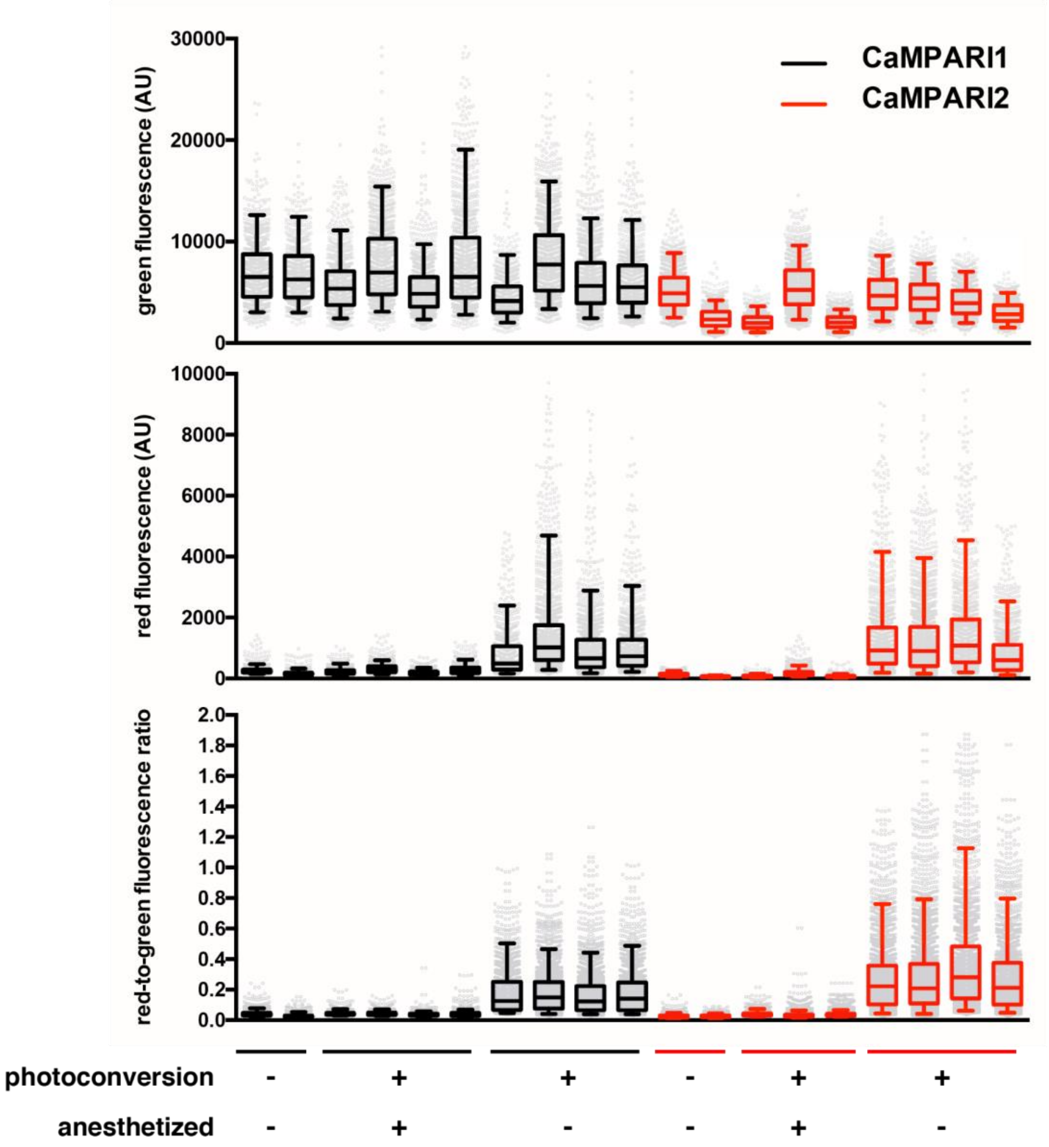

Supplementary Figure 10: Boxplots representing the per-cell green and red fluorescence and red-to-green fluorescence ratio in individual zebrafish. Each boxplot represents one fish. Grey dots are individual cells. Box represents 1st, 2nd and 3rd quartile while whiskers indicate 5th and 95th percentile.

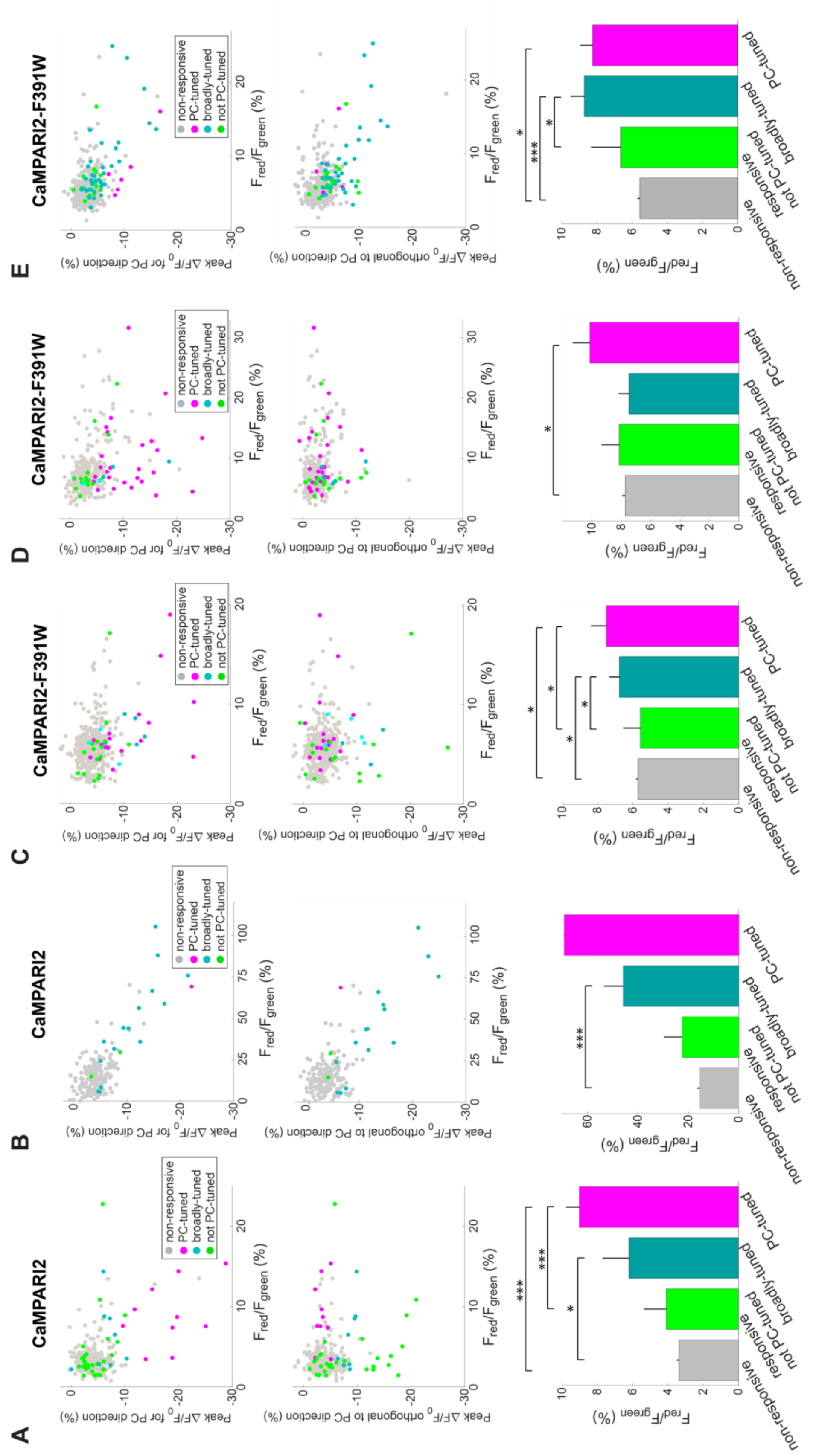
A
B
C
D
E
CaMPARI2
CaMPARI2
CaMPARI2-F391W
CaMPARI2-F391W
CaMPARI2-F391W
non-responsive
PC-tuned
broadly-tuned
not PC-tuned
Peak ΔF/F0 for PC direction (%)
Peak ΔF/F0 orthogonal to PC direction (%)
Fred/Fgreen (%)
non-responsive
responsive not PC-tuned
broadly-tuned
PC-tuned

Supplementary Figure 11: Comparison of CaMPARI2 and CaMPARI2-F391W activity and PC in the mouse primary visual cortex. (**A-B**) Summary of CaMPARI2 performance in the mouse visual cortex. Upper panel: Correlation between peak $\Delta F/F_0$ response for the northward moving grating stimulus and the red-to-green ratio for individual cells. Middle panel: Similar comparison for the eastward moving grating stimulus (orthogonal to the PC direction shown in the upper panel). Lower panel: comparison of red-to-green ratio of PC-tuned cells, not PC-tuned cells, broadly-tuned cells and cells that were not identified as responsive. (**C-E**) Summary of CaMPARI2-F391W performance in the mouse visual cortex, panels show the same comparison as in panel **A-B.** For both constructs, PC efficiency was higher for PC-tuned cells, leading to a significant increase in red-to-green ratio. Note that differences between PC-tuned and non-responsive cells are larger for CaMPARI2 than CaMPARI2-F391W. * $p<0.05$, ** $p<0.01$, *** $p<0.001$ (Wilcoxon Ranksum Test). Error bars indicate the standard error.

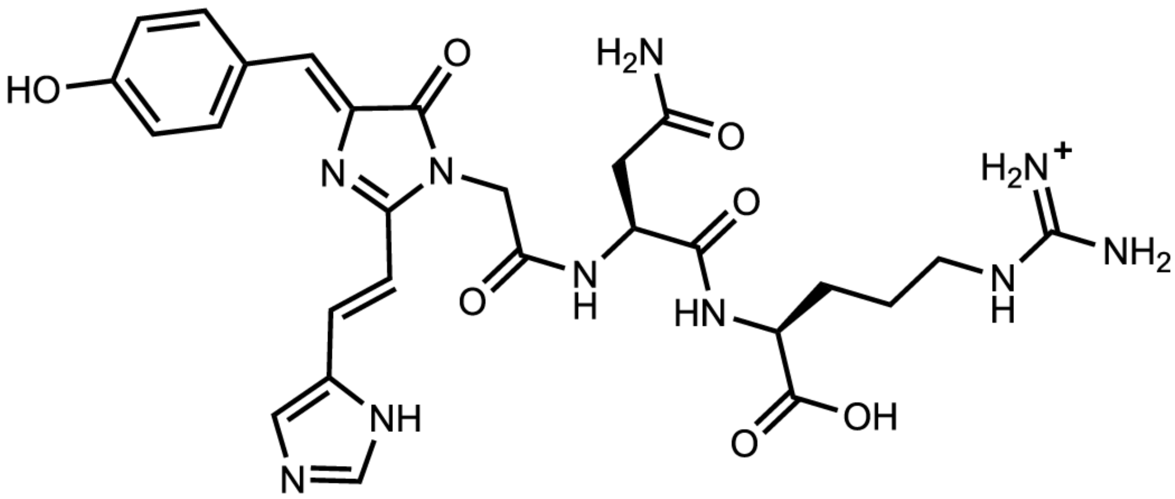

Supplementary Figure 12: Epitope structure. Structure of the proteolytic fragment used to raise the anti-CaMPARI-red antibody, consisting of the red EosFP chromophore and two downstream amino acids (Asn – Arg).

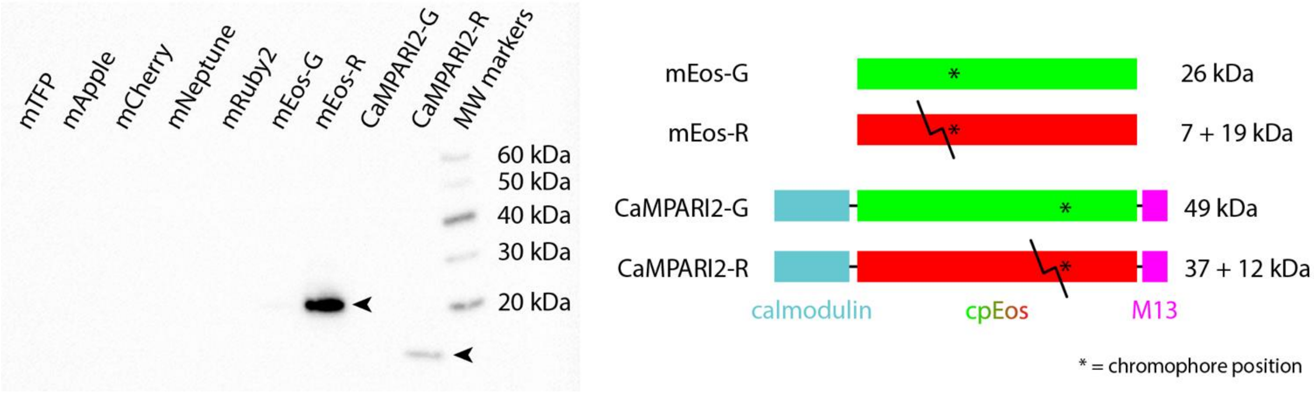

Supplementary Figure 13: Western blot. Western blot of a range of red fluorescent proteins with different chromophore structures, as well as EosFP and CaMPARI2 in the green and red state, probed with the anti-CaMPARI-red monoclonal antibody. All red fluorescent proteins are ±26 kDa and the expected molecular masses of EosFP and CaMPARI2 in the green and red state are shown in the right-hand panel. Each well was loaded with 0.5 µg of protein.

Heavy chain (DNA)

```
  1 ATGGCTGTCC TGGGGCTGCT TCTCTGCCTG GTGACGTTCC CAAGCTGTGT
051 CCTGTCCCAG GTGCAGCTGA AGGAGTCAGG ACCTGGCCTG GTGGCACCCT
101 CACAGAGCCT GTCCATCACA TGCACTGTCT CTGGGTTCTC ATTATCCAGA
151 TATAGTGTAC ACTGGGTTCG CCAGCCTCCA GGAAAGGGTC TGGAATGGCT
201 GGGATTCATA TGGGGTGTTG GATCCACAGA CTATAATTCA GCTCTCAGGT
251 CCAGACTGAC CATCACCAAG GACAACTCCA AGAGCCAGGT TTTCTTAAAA
301 ATGAACAGTC TGCAAACTGA TGACACAGCC ATGTACTACT GTGCCAGAAG
351 CCCGATCTAC TATGATTACG ACGCCTCCTA TGCTATGGAC TACTGGGGTC
401 AAGGAACCTC AGTCACCGTC TCCTCA
```

Heavy chain (protein)

Leader sequence-FR1-CDR1-FR2-CDR2-FR3-CDR3-FR4

```
  1 MAVLGLLLCL VTFPSCVLSQ VQLKESGPGL VAPSQSLSIT CTVSGFSLSR
 51 YSVHWVRQPP GKGLEWLGFI WGVGSTDYNS ALRSRLTITK DNSKSQVFLK
101 MNSLQTDDTA MYYCARSPIY YDYDASYAMD YWGQGTSVTV SS
```

Light chain (DNA)

```
  1 ATGAAGTTGC CTGTTAGGCT GTTGGTGCTG ATGTTCTGGA TTCCTGCTTC
 51 CAGCAGTGAA GTTTTGATGA CCCAAACTCC ACTCTCCCTG CCTGTCAGTC
101 TTGGAGATCA AGCCTCCATC TCTTGCAGGT CTAGTCAGAG CATTGTACAT
151 AGTAATGGAA ACACCTATTT AGAGTGGTAT CTGCAGAAAC CAGGCCAGTC
201 TCCAAAGCTC CTGATCTACA AAGTTTCCAA CCGATTTTCT GGGGTCCCAG
251 ACAGGTTCAG TGGCAGTGGA TCAGGGACAG ATTTCACACT CAAGATCAGT
301 AGAGTGGAGG CTGAGGATCT GGGAGTTTAT TACTGCTTTC AAGGTTCACA
351 TGATCCGTGG ACGTTCGGTG GAGGCACCAA GCTGGAAATC AAA
```

Light chain (protein)

Leader sequence-FR1-CDR1-FR2-CDR2-FR3-CDR3-FR4

```
  1 MKLPVRLLVL MFWIPASSSE VLMTQTPLSL PVSLGDQASI SCRSSQSIVH
051 SNGNTYLEWY LQKPGQSPKL LIYKVSNRFS GVPDRFSGSG SGTDFTLKIS
101 RVEAEDLGVY YCFQGSHDPW TFGGGTKLEI K
```

Supplementary Figure 14: Anti-CaMPARI-red antibody sequence. Sequence analysis of the variable regions of the anti-CaMPARI-red antibody.

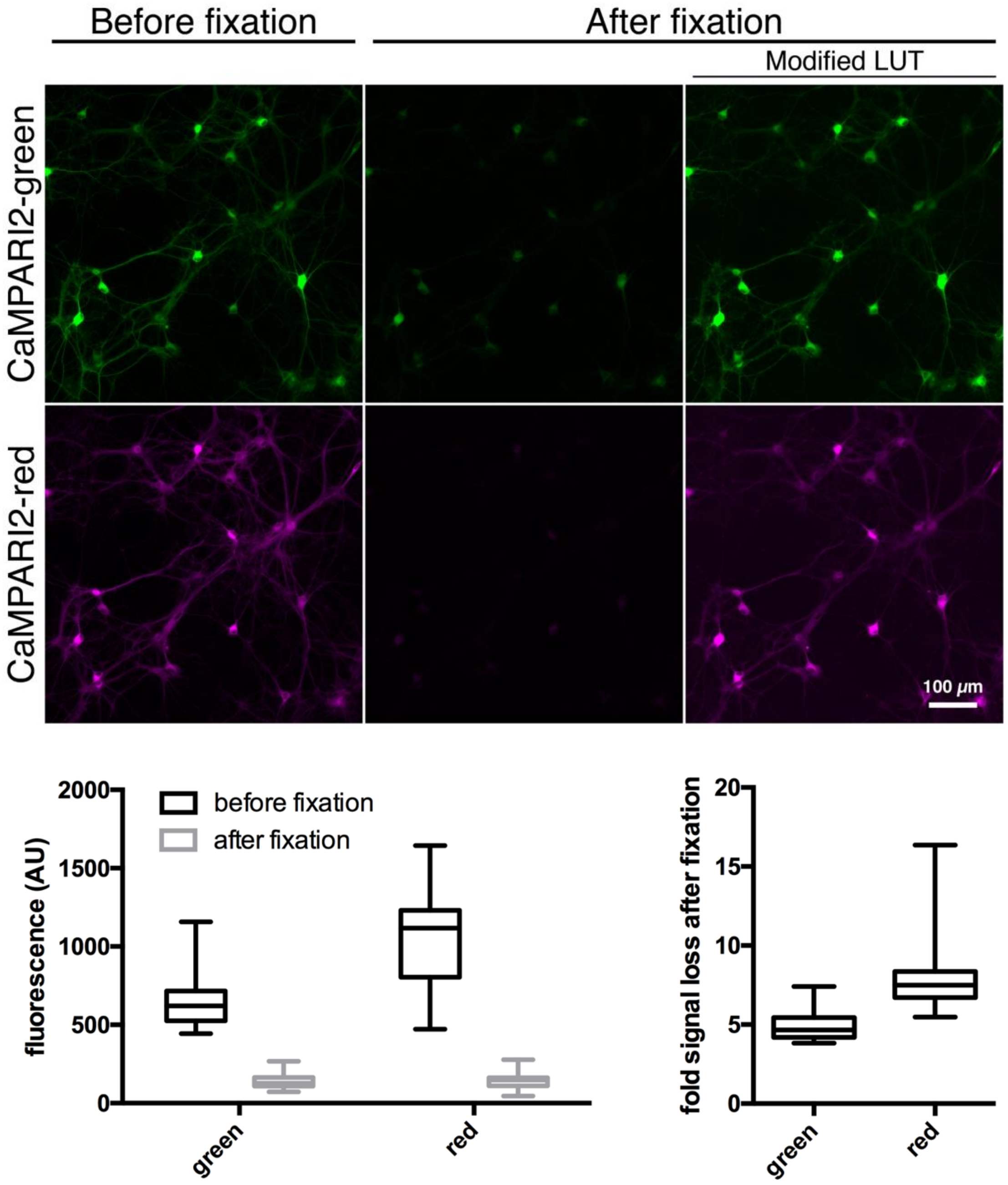

Supplementary Figure 15: Decrease of per-image green and red fluorescence signal of CaMPARI2 in primary neurons after formaldehyde fixation. Representative images of CaMPARI2-expressing primary rat hippocampal neurons after photoconversion. Left, before fixation; middle and right; after fixation (10 min with 4% PFA in PBS-EGTA at room temperature). The right column has a modified color lookup table (LUT) to show the preserved overall structure of the neurons after fixation. Boxplots represent the decrease in green and red fluorescence of 31 images (each containing >20 neurons) before and after fixation. Box represents 1st, 2nd and 3rd quartile while whiskers represent the minimum and maximum value.

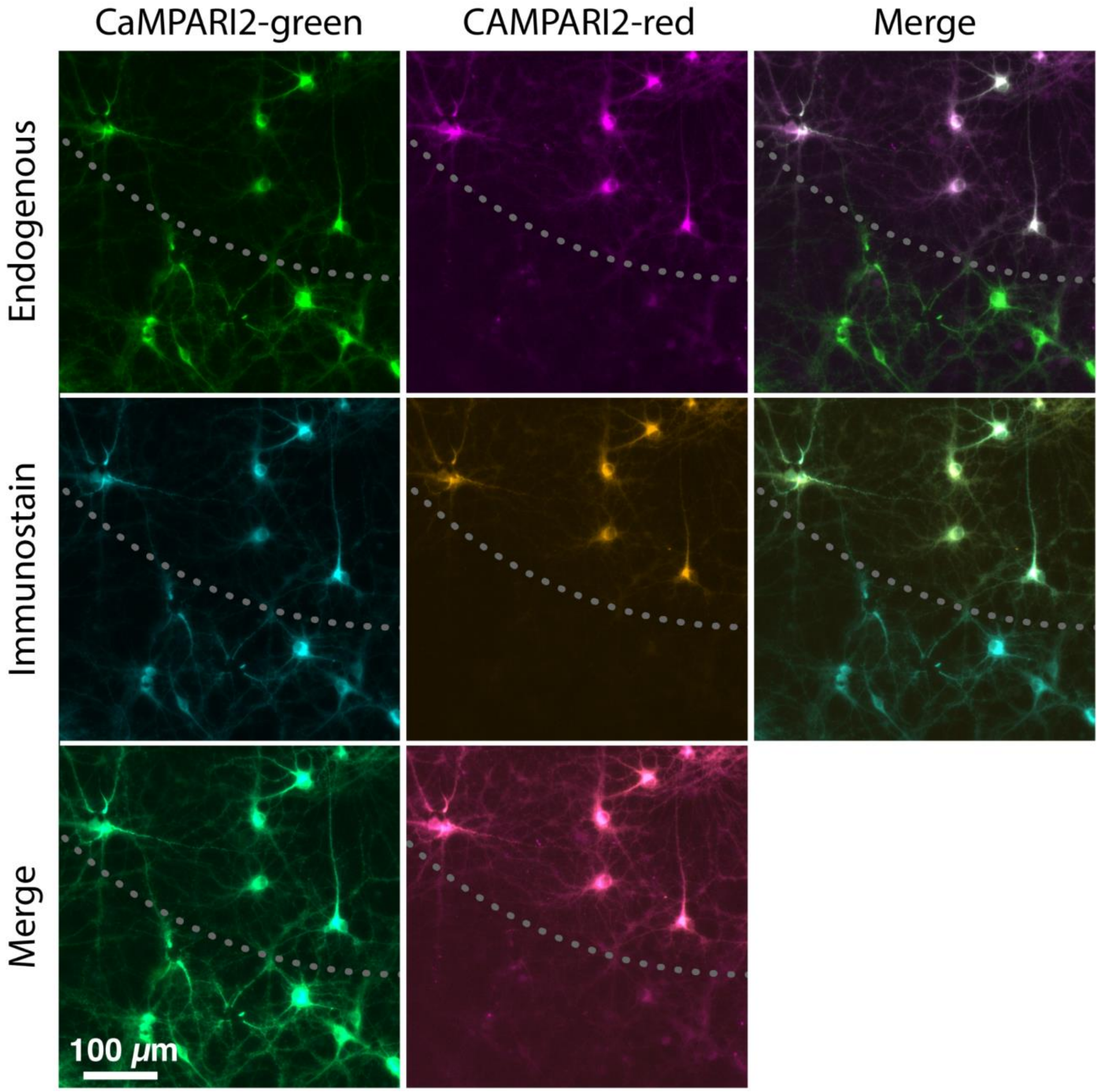

Supplementary Figure 16: Immunohistochemical staining of cultured neurons with anti-CaMPARI-red antibody. The green and red fluorescence of CaMPARI2 after fixation are shown in green and magenta, respectively, while the anti-FLAG antibody signal is shown in cyan and the anti-CaMPARI-red antibody signal is shown in orange. The dotted line represents the edge of the illumination spot for photoconversion (photoconversion above, no photoconversion below). Right and bottom panels show composites of the images to the left or above.

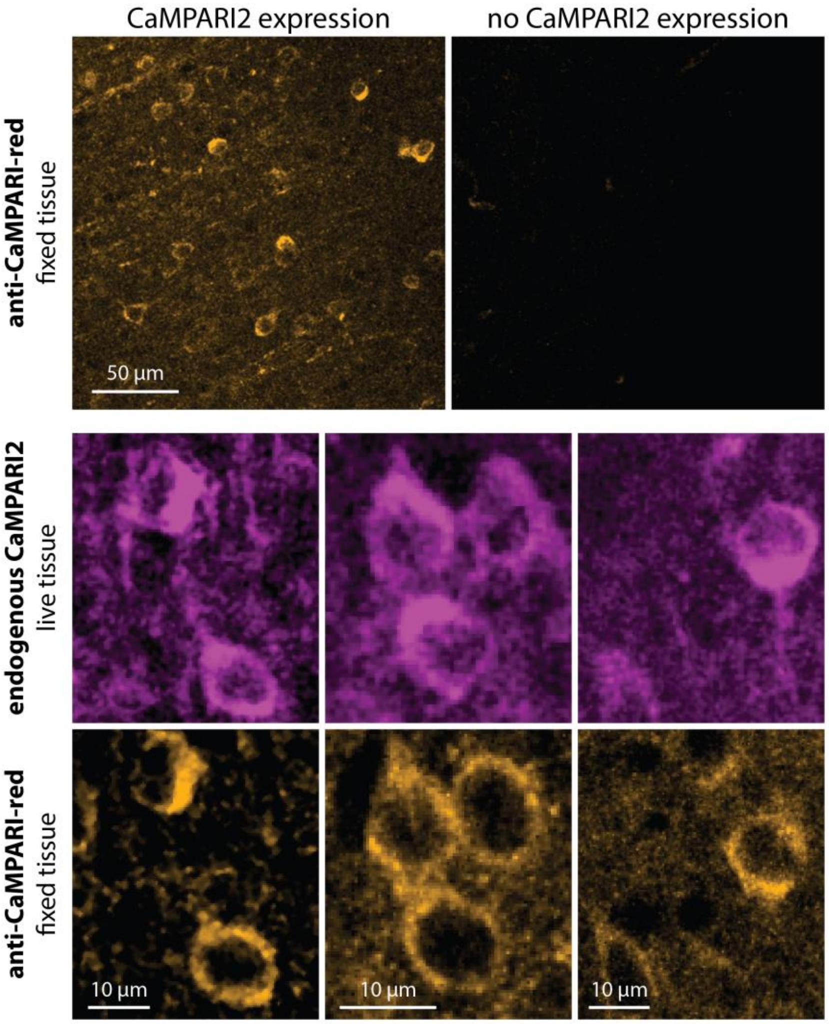

Supplementary Figure 17: Mouse tissue immunostain. Mouse brain tissue expressing CaMPARI2 was photoconverted in the presence of KCl, fixed with paraformaldehyde and immunostained. The anti-CaMPARI-red antibody did not stain the hemisphere contralateral to the injection side (top). The anti-CaMPARI-red antibody signal overlaps well with the endogenous CaMPARI2 signal, imaged before fixation (bottom).

Supplementary Tables

mutation	ΔF/F	Max green (AU)	Max red (AU)	ΔP/P	K_d (nM)
CaMPARI1 (control)	4.27± 0.57	165± 46	276± 90	23.16± 3.10	212
Q142V	4.90	186	367	36.29	207
M143L	4.20	164	307	32.55	283
A146L	2.78	103	170	40.49	372
F198Y	3.63	219	455	30.74	219
C202T	8.86	169	298	27.67	268
C202A	4.63	185	290	31.12	243
L217I	4.33	241	485	34.08	240
L252V	3.65	224	433	36.26	232
N345S	3.61	253	266	36.05	235

Supplementary Table 1: *In vitro* characteristics of the CaMPARI1_W391F-V398L single amino acid mutation library hits. The 9 most interesting hits from the CaMPARI1_W391F-V398L single amino acid site saturation mutagenesis libraries are shown. **ΔF/F** is the difference in green fluorescence intensity between the calcium-bound and calcium-free state divided by the fluorescence of the calcium-bound state of CaMPARI. **Max green / max red** is the green/red fluorescence intensity of the calcium-free state of CaMPARI. **ΔP/P** is the difference in extent of photoconversion between the calcium-bound and calcium-free state of CaMPARI divided by the extent of photoconversion of the calcium-bound state. Higher numbers thus reflect a higher photoconversion contrast ratio. **K_d** is the equilibrium constant of calcium binding determined by measuring the green fluorescence in different Ca^{2+} buffers. CaMPARI1 was used as a control in each 96-well block of the library and the average value of those recordings are reported here with standard deviation. Color coding is such that the more desirable values are darker.

Q142	M143	A146	F198	C202	L217	L252	N345	ΔF/F	Normalized max green (AU)	Normalized max red (AU)	ΔP/P	Kd (nM)
**CaMPARI1 (control)**								**2.98 ± 0.27**	**1 ± 0.056**	**1 ± 0.10**	**1 ± 0.12**	200
V	L		Y	T	I		S	2.75	1.67	1.72	4.97	414
V	L		Y	T			S	2.00	1.83	1.93	4.11	415
V			Y	T	I		S	2.82	1.51	1.73	3.86	285
	L		Y	T	I		S	2.11	1.79	1.80	4.17	440
V	L		Y		I		S	3.09	1.40	1.55	4.45	325
V	L		Y	A	I			3.37	1.24	1.90	3.99	329

Supplementary Table 2: ***In vitro*** **characteristics of the CaMPARI1_W391F-V398L combinatorial library hits.** The 6 most interesting hits from the CaMPARI1_W391F-V398L combinatorial library lysate screens are shown. **ΔF/F** is the difference in green fluorescence intensity between the calcium-bound and calcium-free state divided by the fluorescence of the calcium-bound state of CaMPARI. **Normalized max green / max red** is the green/red fluorescence intensity of the calcium-free state of CaMPARI normalized to the EBFP signal relative to CaMPARI1. **ΔP/P** is the difference in extent of photoconversion between the calcium-bound and calcium-free state of CaMPARI divided by the extent of photoconversion of the calcium-bound state relative to CaMPARI1. Higher numbers thus reflect a higher photoconversion contrast ratio relative to CaMPARI1. **K_d** is the equilibrium constant of calcium binding determined by measuring the green fluorescence of purified protein in different Ca^{2+} buffers. CaMPARI1 was used as a control in each 96-well block of the library and the average value of those recordings are reported here with standard deviation. Color coding is such that the more desirable values are darker. CaMPARI2 (without epitope tags) is in a red box.

	ΔF/F	K_d (nM)	Hill coefficient
**CaMPARI2 no tags**	8.0	267	2.8
**F391W**	6.2	145	3.3
**G395A**	7.0	173	3.2
**F391W-L398V**	6.7	174	3.2
**T394A**	10.4	196	3.0
**S387L**	5.0	346	2.9
**L398V**	5.9	376	3.4
**F391L**	6.2	525	2.9
**H396K**	9.1	548	2.6
**F391M**	5.2	671	3.1
**F391W-L398T**	5.9	720	2.9
**F391Y**	4.6	801	2.6
**F391V**	4.5	806	3.2
**F391W-G395D**	4.9	865	2.8
**L398T**	5.4	1404	2.8
**T394D**	3.5	2107	2.1

Supplementary Table 3: Full list of CaMPARI2 (without tags) affinity mutants designed. CaMPARI2 (no tags) was used as template for 15 affinity mutants. Shaded mutants were selected and further characterized (see Table S4). Note that the mutations mentioned here are relative to CaMPARI2, which contains the W391F-V398L mutations compared to CaMPARI1.

	λ_{ex}, G (nm)	λ_{em}, G (nm)	λ_{ex}, R (nm)	λ_{em}, R (nm)	ε, G (mM^{-1} cm^{-1})	ε, R (mM^{-1} cm^{-1})	QY, G (%)	QY, R (%)	Brightness, G^a	Brightness, R^a	K_d Ca^{2+} (nM) (fluorescence)	ΔF / F^b	K_d Ca^{2+} (nM) (photoconversion)	k_{off} (s^{-1})	k_{on} (s^{-1})
**CaMPARI1**	498	514	560	576	73.5 ± 5.8	32	78	58	1.0	1.0	134.7 ± 10.8	5.9 ± 0.2	100	0.29 ± 0.0044	57.5 ± 1.6
**CaMPARI2**	502	516	562	577	111.3 ± 2.2	65	81	65	1.6	2.3	199.2 ± 11.8	7.8 ± 1.8	224	0.91 ± 0.067	53 ± 0.98
**CaMPARI2 _F391W**	502	516	562	577	114.8 ± 1.7	60	81	62	1.6	2.0	109.7 ± 2.7	6.8 ± 0.3	141	0.48 ± 0.0029	85 ± 1.1
**CaMPARI2 _H396K**	502	516	562	577	117.9 ± 10.1	58	85	65	1.7	2.0	356.7 ± 26.1	6.4 ± 1.1	389	1.8 ± 0.019	27 ± 4.5
**CaMPARI2 _F391W-G395D**	502	516	562	577	122.1 ± 6.1	61	86	64	1.8	2.1	530.0 ± 39.6	5.7 ± 0.6	546	1.3 ± 0.075	31 ± 0.25
**CaMPARI2 _L398T**	502	516	562	577	114.2 ± 3.6	58	80	66	1.6	2.1	824.6 ± 26.2	5.4 ± 0.8	828	2.1 ± 0.012	75 ± 1.1
**CaMPARI2 _F391W-L398V** no epitope tags	503	515	562	577	91.8	44	81	67	1.3	1.6	174.2 ± 29.3	6.2 ± 1.4	NM	0.37 ± 0.0028	66 ± 1.3

Supplementary Table 4: Photophysical properties of CaMPARI1, CaMPARI2 and 4 affinity variants of CaMPARI2. ε is the extinction coefficient in $mM^{-1}cm^{-1}$. [a]Brightness is expressed as molecular brightness (extinction coefficient × quantum yield) relative to the molecular brightness of CaMPARI1 in the corresponding state. [b]$\Delta F/F$ is the difference in green fluorescence intensity between the calcium-bound and calcium-free state divided by the fluorescence of the calcium-free state of CaMPARI. NM = not measured. Errors represent the standard deviation of repeated measurements.

	CaMPARI1			CaMPARI2			ratio
	+ Ca^{2+} / + stimulation (s^{-1})	- Ca^{2+} / - stimulation (s^{-1})	Rate contrast	+ Ca^{2+} / + stimulation (s^{-1})	- Ca^{2+} / - stimulation (s^{-1})	Rate contrast	
Protein	0.020	0.0010	20	0.026	0.00022	120	6.0
Neurons	0.22	0.023	10	0.14	0.0046	30	3.0
Slices†	0.40	0.0097	41	0.22	0.0021	105	2.6

Supplementary Table 5: Rate contrast of CaMPARI1 and CaMPARI2 in different *in vitro* and *ex vivo* settings. The apparent rate of photoconversion in purified protein, cultured rat hippocampal neurons and rat hippocampal slice cultures is given for high calcium / stimulated and low calcium / unstimulated (protein / neurons and cultured slice, respectively) conditions. The rate contrast is calculated as the ratio of photoconversion in high calcium / with stimulation to photoconversion in low calcium / without stimulation. The final column "ratio" is rate contrast of CaMPARI2 divided by the rate contrast of CaMPARI1 for each preparation. †The slice culture experiments (data shown in Figure 2) used CaMPARI2_F391W-L398V (no epitope tags) instead of CaMPARI2.

Supplementary Methods

Characterization of purified CaMPARI proteins

- Protein expression and purification

pRSet-NES-His_6-CaMPARI was transformed in T7express cells and grown in auto-induction medium[2] at 30˚C for 40 h and kept at 4˚C for 32 h before harvesting. The pellet was lysed through two freeze-thaw cycles, the lysate was next diluted in 10 ml B-PER (Thermo Scientific), incubated for 1 h at 30˚C and finally sonicated for 1 minute on ice. The lysate was cleared by spinning it for 30 minutes at 5000 g and NiNTA agarose resin (Qiagen) was added to the supernatant. The resin was loaded on a column, washed with 10 column volumes of TBS buffer (19.98 mM Tris, 136 mM NaCl, pH 7.4) and 10 column volumes TBS with 10 mM imidazole. Finally, the protein was eluted in TBS buffer containing 200 mM imidazole. Proteins were stored at 4˚C.

- Steady-state spectroscopic characterization

Absorption spectra were measured using a Cary 100 UV-VIS spectrophotometer (Agilent). Excitation and emission spectra of the green and red form were acquired using a Cary Eclipse fluorimeter (Agilent). For the green form, excitation/emission wavelength was set at 460/550 nm and the emission/excitation scanned from 470-720/250-540 nm. For the red form, excitation/emission wavelength was set at 530/620 nm and the emission/excitation scanned from 540-720/250-610 nm.

The extinction coefficients of the green form (at 500 nm) were determined using Ward's method,[3] comparing the absorption of identical dilutions of protein in TBS buffer at pH 7.4 and phosphate buffer at pH 13, assuming an extinction coefficient of 44000 $cm^{-1}M^{-1}$ at 440 nm for the base-denatured protein. For the red form, a sample of protein was split in two, one of which was (partially) photoconverted to the red form. From the green extinction coefficient at 500 nm and the difference in green absorption (at 500 nm) and red absorption (at 560 nm), the extinction coefficient of the red species was determined. These measurements all took place in the absence of free Ca^{2+}.

The quantum yield of both the green and red form, in the absence of free Ca^{2+}, was measured using an absolute quantum yield spectrometer (Quantarus-QY C11347, Hamamatsu) set at an excitation wavelength of 480/560 nm for the green/red species.

- Dissociation constant and photoconversion rate

To measure the K_d of calcium binding and free Ca^{2+}-dependent photoconversion efficiency, we diluted protein in 12 different MOPS-KCl buffers (50 mM MOPS, 100 mM KCl, pH 7.2) with EGTA-buffered free Ca^{2+} concentrations between 1 nM and 39 µM. The green fluorescence emission (excitation 495 nm, emission at 520 nm) was measured on an Infinity M1000 fluorescence plate reader (Tecan) to obtain a titration curve which was fitted to Supplementary Equation (1) (Prism, Graphpad) to obtain the K_d.

To assess the photoconversion efficiency, the protein was similarly diluted in 12 Ca^{2+} buffers, split into 18 identical aliquots and irradiated with 405 nm LED light (200 mW/cm^2, Loctite) for 0-1-2-3-5-10-15-20-25-30-45-60-90-120-150-180-240-300 s. Before measuring red fluorescence emission, all free Ca^{2+} was chelated by adding a saturating amount of EGTA to all samples. The red fluorescence emission (excitation 555 nm, emission at 580 nm) was measured on an Infinity M1000 fluorescence plate reader (Tecan). For each calcium concentration, the red fluorescent signal was fitted to an exponential function to extract the apparent rate constant of photoconversion. At higher photoconversion times, and most notably at and above 150 s of photoconversion, the curves started to show a downward trend, which we attribute to photobleaching. To avoid this artifact from influencing the fits, we left out the last few data points for each curve. From these fits, we determined a global plateau value by averaging the plateau value of the three highest calcium concentrations and repeated, for each sample, the fits with the plateau value fixed to this value. The rate constants were then plotted against the calcium concentrations. We noticed that at the higher calcium concentrations, the rate constants dropped from the maximum value, especially for the CaMPARI1 samples. We therefore left out these values before fitting the data to Supplementary Equation (2) (IgorPro, Wavemetrics) to extract an apparent K_d.

Supplementary Equation (1) $$y = \Delta F \cdot \left(1 - \frac{x^h}{{K_d}^h + x^h}\right) + F_{min}$$

Supplementary Equation (2) $$y = \Delta F \cdot \left(\frac{x^h}{{K_d}^h + x^h}\right) + F_{min}$$

with $y =$ fluorescent signal (AU)

$K_d =$ dissociation constant in M

$h =$ Hill coefficient

$x =$ [Ca^{2+}] in M

- Kinetics

The rates of calcium binding (k_{on}) and unbinding (k_{off}) were determined by stopped flow. Proteins were diluted in TBS buffer with 10 µM EGTA / $CaCl_2$. Using a SX-20 (Applied Photophysics) with 490 nm LED, the protein solution was quickly mixed with a 10 mM $CaCl_2$ / EGTA solution in TBS and green fluorescence was followed in time. The decrease / increase in fluorescence was fitted with a bi-exponential curve to retrieve the rate constants using IgorPro (Wavemetrics).

- Two photon action cross-sections

CaMPARI2 two-photon action cross-sections were measured in the green and red state and in saturating EGTA and saturating Ca^{2+} conditions. The protein samples were placed on an inverted microscope equipped with a 60× 1.2NA objective and excited with Ti:Sapphire (1 mW, 735-1075 nm) or OPO laser (2 mW, 1000-1250 nm). Emission was collected through a 720SP

and 550/88 filter (green form) or a 720SP and 607/70BP filter (red form). The CaMPARI2 two-photon excitation spectra (average of 2 (high Ca^{2+}) or 4 (low Ca^{2+}) runs) were measured side-by-side with fluorescein (in borate buffer, pH 9.7) and rhodamine B (in methanol) as references for the action cross-section.[1] Emission in the red state was corrected for differential detection efficiency between rhodamine B and CaMPARI2-red due to the emission filters used and the respective emission spectra.

CaMPARI characterization in primary neuron cultures

Primary rat hippocampal neurons were prepared as described previously[4] and infected with AAV2/1 virus encoding CaMPARI under control of the human synapsin promoter 3 days after isolation. 6 days after infection, the coverslips with neurons were transferred to a 24-well plate with neuron imaging medium containing spontaneous synaptic activity-blocking drugs (described previously[5]). A custom-built electrode, controlled by a high current isolator (A385, World Precision Instruments) set at 90 mA was inserted into the medium for field stimulation. The neurons were imaged on a wide-field microscope (Nikon Ti Eclipse) equipped with a LED illuminator (SPECTRA-X, Lumencor), a 20× objective and an sCMOS camera (Zyla, Andor).

- Photoconversion in cultured primary neurons

To follow photoconversion in time, we irradiated the CaMPARI-expressing neurons with 0.5 s of 395 nm light (~3 W/cm^2) accompanied by 40 field pulses of 1 ms at 80 Hz or in the absence of field stimulation. After 30 s in the dark (to allow the calcium to fully unbind from CaMPARI), the cells were imaged in the green and red channels. This procedure was repeated 30 times, to result in 15 s of accumulated 395-nm light irradiation. The resulting red fluorescence was plotted in time, and fitted with an exponential function to extract the rate constant of photoconversion.

- Calcium unbinding kinetics in cultured primary neurons

To measure the rate of calcium unbinding in CaMPARI-expressing neurons, we continuously imaged the green channel at relatively low light power (270 µW/cm^2) to avoid photobleaching and field stimulated the neurons (1 ms pulses at 80 Hz), followed by 30 s of recovery. We delivered first 1 pulse, then 2, 5, 10, 20, 40 and 80 pulses. The fluorescence recovery was fitted with an exponential function and averaged over the 150-200 cells in total (from 3 trials in 2 independent culture dishes) to extract the rate constant of calcium unbinding. Generally, the traces resulting from one field pulse could not be fitted, and the two and five pulse traces could only be fitted in 20-50% of neurons.

- Photoconversion in primary neurons at different stimulation frequencies

To observe the effect of the affinity tuning on the photoconversion in CaMPARI-expressing neurons, we imaged the pre-green and pre-red signal, field stimulated the neurons for 2 s at 0 (no stimulation), 2, 10, 30 or 80 Hz while photoconverting using ~3 W/cm^2 of 395 nm light, and measured a post-green and post-red image again. From the post-red image, the pre-red image

was subtracted. An image mask was calculated based on the pre-green and the background-corrected red image was divided by the pre-green signal in the masked area. The ratio between the stimulated samples and non-stimulated sample was calculated, resulting in a fold increase in red-to-green ratio upon stimulation.

- Delay between stimulation and photoconversion light

We stimulated several fields of view of neuron cultures expressing CaMPARI variants with 20 field pulses at 80 Hz and 250 ms of photoconversion light. 45 s later, green and red fluorescence images were acquired. This stimulation and photoconversion was repeated for 10 cycles. This protocol was performed with 0, 0.5, 1, 2, 5 and 30 s delay between stimulation and photoconversion as well as a negative control without stimulation. For each, the rate of photoconversion was measured as the slope of the increase of red/green signal vs cumulative photoconversion time. This was normalized to 1 at the highest rate. For comparison, calcium binding and fluorescence change kinetics were measured by continuous imaging of the green CaMPARI fluorescence during an equivalent stimulation of 20 field pulses at 80Hz.

CaMPARI2-red signal turnover experiments:

CA1 neurons in rat organotypic hippocampal slice cultures were electroporated at DIV15 with DNA (20ng μl^{-1}) encoding CaMPARI2_F391W-L398V (no epitope tags). Four days later, two-photon z-stacks were collected using two MaiTai Deep See lasers. For each z-plane, two frames (images) were acquired, exciting at 980 nm (2-5 mW measured at the back-focal plane of the objective) and 1040 nm (3-8 mW), respectively. The procedure was repeated several times (up to 3 days). Between imaging sessions slices were put back in the incubator. To increase intracellular calcium in response to synaptic activity, a monopolar electrode was placed in the stratum radiatum and 0.2 ms electrical pulses were delivered 100 times at 100 Hz at an intensity that induced postsynaptic spiking in a neighboring nontransfected CA1 neuron. UV light (395 nm, 16 mW mm^{-2}) was applied for 2 s with a 1 s delay relative to the start of stimulation (24 neurons, 4 slices). Images were taken at time points t = -0.5, 0, 6, 12, 24, 30, 48, 60, 72 and 72.5 hours with t = 0 immediately after stimulation + UV light or UV light alone. To compare intensity between imaging sessions, images were normalized to a calibration solution containing 200 $\mu g\ ml^{-1}$ fluorescein and 100 $\mu g\ ml^{-1}$ sulforhodamine 101. These higher dye concentrations were selected to closely match the intensity of the CaMPAR2 green species prior to photoconversion, and the red species after photoconversion. Fluorescence values of the CaMPARI green and red channels were divided by the corresponding calibration values. R/G is the normalized red fluorescence divided by the normalized green fluorescence.

Macro for 2P image analysis of CaMPARI conversion in neurons

We stablished a workflow to analyze 3D images generated in ScanImage[6] employing alternate-frame 2P 980/1040nm wavelength acquisition. We used Fiji[7], an image processing package distribution of ImageJ. Channels corresponding to 980 nm or 1040 nm acquisition were deinterleaved and 3D stitched using the Pairwise Stitching plugin[8]. For further analysis, we used

the green fluorescence collected at 980 nm excitation and red fluorescence collected at 1040 nm excitation. After median filtering and rolling ball background subtraction[9], a maximum intensity projection image was generated and saved. Maximum pixel values from ROIs drawn at neuronal somata were saved in Excel and divided from fluorescence values obtained from median-filtered fluorescein/SR101 solutions. Code of the Fiji/ImageJ macro is reproduced below.

```
//This is a macro for analysis of CaMPARI 4ch images acquired with alternate-frame 2P 980/1040nm wv acquisition in ScanImage
//It deinterleaves, corrects for chromatic aberration, filters image, makes maximum projection, subtracts background and finally
//saves the image containing the relevant channels for analysis (ch1/ch4) APA 26.3.18

run("Bio-Formats Windowless Importer", "view=Hyperstack stack_order=XYCZT");
imageTitle=getTitle();
run("Deinterleave", "how=4");
run("Merge Channels...", "c1=["+imageTitle +" #1] c2=["+imageTitle +" #2] create");
rename("Composite 980.tif");
run("Merge Channels...", "c3=["+imageTitle +" #3] c4=["+imageTitle +" #4] create");
rename("Composite 1040.tif");
wait(100);
run("Pairwise stitching", "first_image=[Composite 980.tif] second_image=[Composite 1040.tif] fusion_method=[Overlay into composite image] fused_image=[Composite 980.tif<->Composite 1040.tif] check_peaks=5 compute_overlap x=0 y=0.0000 z=0.0000 registration_channel_image_1=[ Average all channels] registration_channel_image_2=[ Average all channels]");
selectWindow("Composite 980.tif");
close();
selectWindow("Composite 1040.tif");
close();
imageTitle=getTitle();
run("Split Channels");
run("Merge Channels...", "c1=[C1-Composite 980.tif<->Composite 1040.tif] c4=[C4-Composite 980.tif<->Composite 1040.tif] create");
selectWindow("C3-Composite 980.tif<->Composite 1040.tif");
close();
selectWindow("C2-Composite 980.tif<->Composite 1040.tif");
close();
Stack.setChannel(1);
run("Green");
run("Enhance Contrast", "saturated=0.35");
Stack.setChannel(2);
run("Red");
run("Enhance Contrast", "saturated=0.35");
imageTitle=getTitle();
run("Median...", "radius=0.5");
run("Z Project...", "projection=[Max Intensity]");
run("Subtract Background...", "rolling=100");
saveAs("Tiff");
selectWindow("Composite 980.tif<->Composite 1040.tif");
close();
```

Western blots with anti-CaMPARI-red antibody

Western blots were prepared by loading 0.5 µg of protein in each well of a bis-tris PAGE gel (Novex, Invitrogen). The gel was blotted onto a PVDF membrane (Invitrolon, Invitrogen) and

blocked for 1 h at room temperature in TBS with 0.1% Tween (TBST) and 5% dry non-fat milk powder (TBST-milk). We diluted mouse bleeds (1:1000), hybridoma supernatant (1:10) or primary antibody (1:10 000), in TBST-milk and incubated for 16 h at 4˚C. Then, the membrane was washed three times in TBST and labeled with a HRP-conjugated horse-anti-mouse (Cell Signaling, 7076) diluted 1:3000 in TBST-milk for 1 h at room temperature. The blot was again washed three times with TBST and 3 times with TBS before bioluminescent substrate was added (SuperSignal West Pico, Thermo Scientific) and the blot imaged.

Immunohistochemistry in neuron cultures

For immunostaining, we used neurons 11 days after infection. One focal area was photoconverted with 5 s of 400-nm light (3 mW/cm^2) using the microscope's 20× objective while the whole dish was stimulated at 80 Hz for 2 s concurrent with the illumination. The neurons were then fixed in 4% paraformaldehyde for 10 minutes at room temperature and washed 3 times with PBS-EGTA. To image at this point, we changed the buffer to PBS-EGTA.

For immunohistochemical staining we applied perm/block1 solution (10% normal goat serum, 0.1% Triton X-100 in PBS) for 1 h at room temperature after the PBS-EGTA wash. Then, primary antibody was applied (mouse-anti-CaMPARI-red 1:10 000, rabbit-anti-flag 1:3000), diluted in perm/block1, and left shaking at 4˚C overnight. The neurons were then washed 3 times with PBS-EGTA and secondary antibodies (goat-anti-rabbit-alexa405 and goat-anti-mouse-alexa647) were applied, diluted 1:1000 in perm/block1 solution. After 1 h at room temperature, they were washed 3 times with PBS-EGTA and imaged.

To measure the extent of endogenous fluorescent signal loss due to fixation, we aligned red and green images of a photoconverted field of view before and after fixation but before staining. The average pixel intensity in a region without neurons was subtracted from the average pixel intensity of the whole field of view. We did this for 30 images of 12 different wells of cultured neurons.

CaMPARI2 *ex vivo* photoconversion and immunostain in mouse acute brain slice

- Viral injections

P21–P25 mice (C57/BL6J, supplied by the Charité Universitätsmedizin Berlin FEM) were deeply anesthetized with ketamine/xylazine (7 mg kg^{-1}/1 mg kg^{-1}), and then injected with 100–200 nl of the adeno-associated virus AAV2/1-Syn-CaMPARI2 in the somatosensory cortex. We waited for > 14 days before use.

- Slice preparation

Coronal slices, 300 µm thick, were prepared from AAV2/1-Syn-CaMPARI2-injected mice (postnatal ages P36–P50). The artificial cerebrospinal fluid (ACSF) used for recordings and brain slicing contained (in mM): 125 NaCl, 2.5 KCl, 1.25 NaH_2PO_4, 25 $NaHCO_3$, 2 $CaCl_2$, 1 $MgCl_2$ and 25

d-glucose, pH ~7.4. Immediately following slice preparation, the slices were incubated at 32°C for 5 min in a solution containing (in mM): 110 choline chloride, 2.5 KCl, 1.25 NaH_2PO_4, 26 $NaHCO_3$, 11.6 sodium ascorbate, 3.1 sodium pyruvate, 7 $MgCl_2$, 0.5 $CaCl_2$ and 10 d-glucose, pH ~7.4, followed by 25 min at 32°C in ACSF and then at room temperature, before recording in a submersion chamber at 32°C. 5 mM KCl was added to the ACSF during CaMPARI2 conversion to depolarize the neurons in the brain slice. All solutions were saturated with 95% O_2/5% CO_2 and had pH ~7.4.

- Photoconversion and imaging

CaMPARI2 photoconversion was delivered using an X-cite 200 W mercury lamp (Excelitas Technologies, Mississauga, Ontario, Canada) and light guide through a 405/10 bandpass filter (Semrock, FF01-405/10-25). Conversion light intensity was ~ 200 mW cm^{-2}.

A Femto 2D two-photon laser scanning system (Femtonics Ltd, Budapest, Hungary) was equipped with a femtosecond pulsed Chameleon Ti:Sapphire laser (Coherent, Santa Clara, CA, USA). Imaging was controlled by the MATLAB-based MES software package (Femtonics). For CaMPARI red and green fluorescence measurements the laser was tuned to λ = 820 nm. Fluorescence was detected in epifluorescence mode with a water immersion objective (LUMPLFL 60×/1.0 NA, Olympus, Hamburg, Germany). Trans-fluorescence and transmitted infrared light were detected using an oil immersion condenser (Olympus; 1.4 NA). Fluorescence was divided by a dichroic mirror at ~590–600 nm, and green and red signals were filtered using 525/50 and 650/50 bandpass filters, respectively.

- CaMPARI immunohistochemistry

After imaging of the live brain slices, they were fixed in PFA (4 %) at 4˚C overnight. Slices were washed in phosphate buffered solution (PBS) and then blocked in normal goat serum (NGS) and PBS (5 % NGS, 1% Triton in PBS) for two hours at room temperature. Brain slices were then incubated in primary antibody solution (anti-CaMPARI-red, 1:1000) at 4˚C overnight. Slices were washed in PBS before incubation in the secondary antibody solution (Alexa 633, goat anti mouse, Invitrogen A21050, 1:500) for two hours at room temperature. Slices were then washed in PBS and mounted in Glycerol (80% in PBS + 2.5 % DAPCO).

- Confocal imaging

Slices were imaged on a confocal laser scanning microscope (Nikon A1Rsi+) using a 20× air objective (Plan Apo, 0.8 NA, 1.000 WD) and a 647 nm laser. Fluorescence was imaged through a 700/50 bandpass emission filter.

Data analysis

Images were processed in ImageJ / Fiji. Data was analyzed and graphs were plotted using IgorPro (Wavemetrics) or Prism (GraphPad). Figures were made using Illustrator (Adobe).

Reagents

DNA constructs for pAAV_hsyn_CaMPARI2, pAAV_hsyn_CaMPARI2_F391W, pAAV_hsyn_CaMPARI2_H396K, pAAV_hsyn_CaMPARI2_F391W-G395D and pAAV_hsyn_CaMPARI2_L398T are available via Addgene (http://www.addgene.org #101060-#101064). AAV virus can be requested at the University of Pennsylvania Vector Core (http://www.med.upenn.edu/gtp/vectorcore). Tg(elavl3:CaMPARI2)^{jf92} transgenic zebrafish will be deposited to the ZIRC (https://zebrafish.org). Drosophila expressing CaMPARI2 and CaMPARI2-L398T under UAS and LexA promoter in chromosome 2 (su(Hw)attP5), 3 (VK00005) or X (su(Hw)attP8) are available from the authors and at the Bloomington Drosophila Stock Center (https://bdsc.indiana.edu, #78316-#78326).

Supplementary References

1. Makarov, N. S., Drobizhev, M. & Rebane, A. Two-photon absorption standards in the 550-1600 nm excitation wavelength range. *Opt. Express* **16,** 4029–4047 (2008).
2. Studier, F. W. Protein production by auto-induction in high density shaking cultures. *Protein Expr. Purif.* **41,** 207–34 (2005).
3. Ward, W. W. Properties of the Coelenterate green-fluorescent proteins. *Biolumin. Chemilumin. Basic Chem. Anal. Appl. Eds. DeLuca, M. McElroy, WD (Academic Press. Inc., NY)* 235–242 (1981).
4. Wardill, T. J. *et al.* A Neuron-Based Screening Platform for Optimizing Genetically-Encoded Calcium Indicators. *PLoS One* **8,** 1–12 (2013).
5. Chen, T.-W. *et al.* Ultrasensitive fluorescent proteins for imaging neuronal activity. *Nature* **499,** 295–300 (2013).
6. Pologruto, T. A., Sabatini, B. L. & Svoboda, K. ScanImage: Flexible software for operating laser scanning microscopes. *Biomed. Eng. Online* **2,** 1–9 (2003).
7. Schindelin, J. *et al.* Fiji: an open-source platform for biological-image analysis. *Nat. Methods* **9,** 676–682 (2012).
8. Preibisch, S., Saalfeld, S. & Tomancak, P. Globally optimal stitching of tiled 3D microscopic image acquisitions. *Bioinformatics* **25,** 1463–5 (2009).
9. Sternberg. Biomedical Image Processing. *Computer (Long. Beach. Calif).* **16,** 22–34 (1983).
